# Prophylactic High-Dose Gabapentin Reduces Opiate Use during Radiation Therapy for Head and Neck Squamous Cell Carcinoma

**DOI:** 10.3390/cancers15072003

**Published:** 2023-03-28

**Authors:** Michelle L. Qiu, Austin J. Iovoli, Michael Khan, Mark K. Farrugia, Sung Jun Ma, Anurag K. Singh

**Affiliations:** 1 Jacobs School of Medicine and Biomedical Sciences, University at Buffalo, 955 Main Street, Buffalo, NY 14203, USA; 2 Department of Radiation Medicine, Roswell Park Comprehensive Cancer Center, Elm and Carlton Streets, Buffalo, NY 14203, USA

**Keywords:** head and neck cancer, radiation therapy, gabapentin, oral mucositis

## Abstract

**Simple Summary:**

Patients undergoing radiotherapy for head and neck cancer often develop painful mouth sores caused by inflammation called oral mucositis. The use of prophylactic gabapentin to reduce pain and opioid use during treatment in this population is controversial. To investigate this further, we evaluated 480 patients with head and neck cancer treated at our institution with high-dose (3600 mg), moderate-dose (200 to 3200 mg), and no gabapentin and examined the rates of opioid use. We found that patients given prophylactic high-dose gabapentin had reduced opioid use and did not require opioids until later in treatment compared with patients taking a moderate dose or no gabapentin. These findings support the use of prophylactic high-dose gabapentin for head and neck cancer patients undergoing radiotherapy to reduce patients’ reliance on opioids for pain relief.

**Abstract:**

Background: The role of prophylactic high-dose gabapentin for the management of oral mucositis during radiation therapy for head and neck squamous cell carcinoma (HNSCC) remains controversial. Methods: A retrospective cohort analysis was performed on primary HNSCC patients treated at our institution. Kruskal–Wallis and Fisher’s exact tests were used to compare the patients’ baseline characteristics. Multivariate competing risk and logistic regressions were performed to evaluate time to first opioid use and feeding tube placement. Results: In total, 480 consecutive HNSCC patients were included. Within this cohort, 186 patients received 3600 mg gabapentin, 182 received 300 to 3200 mg gabapentin, and 112 received no gabapentin. The time to first opioid use was greater in the 3600 mg group compared with the no gabapentin group (34.3 vs. 23.9 days, *p* < 0.001) and to the 300 to 3200 mg group (28.0 days, *p* < 0.001). The proportion of patients requiring opioids at any point during RT was lower in the 3600 mg gabapentin group compared with the no gabapentin group (31.8% vs. 60.1%, *p* < 0.001) and with the 300 to 3200 mg group (63.8%, *p* < 0.001). Conclusions: Prophylactic use of 3600 mg gabapentin was well tolerated, halved overall opioid use, and delayed the time to first opioid use during radiation therapy.

## 1. Introduction

Over 40% of patients treated with radiation therapy (RT) for head and neck squamous cell carcinoma (HNSCC) require opioids to manage oral mucositis (OM) pain [1]. The use of prophylactic gabapentin has been explored in recent studies as a way to manage OM pain without the use of opiates. Gabapentin is a gamma-aminobutyric acid (GABA)-mimetic compound originally developed as an anti-epileptic agent and subsequently found to be effective in the treatment of chronic pain syndromes, particularly neuropathic pain. The mechanism of gabapentin in treating neuropathic pain is not fully understood but evidence suggests it is linked to antagonism of the N-methyl-D-aspartate (NMDA) receptor and calcium channel blockage [2]. Several studies have demonstrated the efficacy of gabapentin in treating neuropathic pain syndromes, particularly in patients with cancer-induced pain [3].

In the treatment of HNSCC, Ma et al. prospectively showed that escalating doses of gabapentin (900, 2700, and 3600 mg) were well tolerated, effective in reducing opiate use during RT, and did not worsen the need for a feeding tube (FT) [4]. In contrast, other prospective trials have found that patients could not escalate gabapentin beyond 900 mg [5], there was no pain benefit with 1800 mg of gabapentin, and patients on gabapentin had a significantly higher rate of FT placement [6]. Consequently, the tolerability, benefits, and feeding tube rate of dose-escalated gabapentin remain controversial. To address this knowledge gap, we performed an extensive observational cohort analysis to evaluate the prophylactic use of 3600 mg daily of gabapentin for OM management.

## 2. Methods

Our institutional review board approved this single-institution retrospective study of HNSCC patients diagnosed and treated with RT between 2015 and 2022. Eligible patients were (1) 18 years or older, (2) diagnosed with pathologically proven Stage II–IV (American Joint Committee on Cancer 7th edition) HNSCC undergoing definitive or adjuvant intent radiation, (3) completed radiation, and (4) survived at least 3 months upon completion of radiation. This study followed the Strengthening the Reporting of Observational Studies in Epidemiology (STROBE) reporting guidelines.

### 2.1. Treatment

All patients completed a staging workup with computed tomography (CT) of the head and neck with contrast and/or positron emission tomography-computed tomography (PET/CT). All patients who underwent definitive radiation therapy were treated with intensity-modulated radiation therapy (IMRT; 70 Gy/35 fractions to the primary tumor, 56 Gy/35 fractions to elective lymph nodes) with or without concurrent chemotherapy, as previously described [7]. All patients who underwent adjuvant radiation therapy were treated with IMRT with the dose depending on pathologic risk factors (60–66 Gy/30–33 fractions to the post-operative bed, 54 Gy/30–33 fractions to the elective lymph nodes).

Prior to and during treatment, all patients received educational materials and were encouraged regarding oral hygiene, hydration, and nutrition. Patients were encouraged to gargle with a saline/baking soda mouthwash rinse as many times as possible per day (e.g., 20 times) and use a compounded elixir of diphenhydramine, xylocaine, and antacid in a 1:1:1 ratio 4 times per day for pain. Further details of our institutional management of OM have been previously described [8].

The institutional standard for prescribing and escalating all HNSCC patients to 3600 mg daily of gabapentin began on 1 July 2018. All patients on gabapentin before this time point were enrolled on a prospective clinical trial involving gabapentin or managed by a pain clinic. All patients received oral gabapentin, starting at 300 mg daily on Day 1 and gradually escalated by adding 300 mg to the total daily dose (e.g., 300 mg twice a day on Day 2, 300 mg three times a day on Day 3). Gabapentin was titrated up to 1200 mg three times a day over the course of a minimum of 12 days. Prior to the prescription of gabapentin, all patients completed a comprehensive metabolic panel to assess them for adequate renal and hepatic function. For patients with impaired renal function during radiation therapy, their gabapentin doses were adjusted accordingly [9]. Reassessment of renal and hepatic function was performed when indicated based on clinical symptoms. Patients continued on gabapentin for the total duration of RT and were tapered off it once the OM symptoms resolved. Patients were prescribed methadone or hydrocodone as needed for breakthrough pain. After shared decision-making between patients and clinicians, gabapentin was tapered off prior to the completion of RT among patients who reported being unable to tolerate it due to significant side effects or symptom burden.

All study data were collected and managed using Research Electronic Data Capture (REDCap), hosted at Roswell Park Comprehensive Cancer Center [10,11]. Baseline patient demographics and tumor characteristics were collected. As a part of routine institutional practice, patients were evaluated weekly while undergoing RT by the radiation clinical team through a physical exam and patient-reported responses to a modified oral mucositis weekly questionnaire – head and neck cancer (OMWQ-HN) survey [12]. The OMWQ-HN is a valid and reliable survey assessing patients’ well-being and function. The development of severe OM was defined as “quite a lot” or “extreme” reported for the mouth and throat soreness item based on the highest reported OM score during RT. The physical exam included an assessment of the extent of OM, weight changes, and feeding tube usage. During RT, the time to opioid prescription and feeding tube (FT) status were prospectively maintained. Feeding tube placement was performed with a multidisciplinary evaluation of factors including evaluations of the patients’ nutritional and functional status, speech, and swallowing, and shared discussions among patients, family members, caregivers, and physicians. Of note, many patients who received RT prior to 2017 had a FT placed prophylactically due to a previous institutional standard. Thereafter, FTs were no longer placed prophylactically but placed only when needed to maintain nutrition.

### 2.2. Statistics

The primary endpoints of this analysis were tolerance of 3600 mg gabapentin, time to first opioid use, and feeding tube placement. Kruskal–Wallis and Fisher’s exact tests were used to compare the baseline characteristics and the proportion of patients requiring opioids during radiation therapy. With the 3600 mg cohort as a reference, multivariate competing risk and logistic regressions were performed to evaluate the time to first opioid use and feeding tube placement, respectively. Multivariate models were adjusted for baseline characteristics, including age, sex, performance status, body mass index, pretreatment feeding tube placement, primary disease site, staging, and unilateral versus bilateral elective neck radiation. Associations between gabapentin dose and the development of severe oral mucositis were examined with Fisher’s exact test.

Bonferroni correction was used for multiple comparisons (the 3600 mg cohort vs. the no gabapentin cohort; the 3600 mg cohort vs. the 300–3200 mg cohort). All statistical tests were two-sided, and *p*-values lower than 0.025 were considered statistically significant. All analyses were performed using R (version 4.0.3, R Project for Statistical Computing, Vienna, Austria).

## 3. Results

Among 480 consecutively treated patients, 186 patients received 3600 mg gabapentin, 182 received 300 to 3200 mg gabapentin (median dose: 1800 mg), and 112 received no gabapentin. The average age was 62.5 (SD: 9.55), 370 patients were male (77.1%), and 435 patients were White (90.6%). Baseline characteristics were well-balanced (Table 1).

There were 223 patients (46.5%) who were eligible for escalation to 3600 mg gabapentin. Moreover, 221 (86.0%) of ineligible patients were treated prior to 1 July 2018. Other reasons included performance status (3.5%), renal function (1.9%), or external pain management (8.6%). Of the eligible patients, 192 patients (86.1%) tolerated 3600 mg gabapentin through to the completion of RT. Reasons for discontinuation included dizziness/weakness (35.5%), hallucinations/confusion (12.9%), somnolence (12.9%), hospitalization (9.7%), or other reasons (29.0%). Side effects attributed to gabapentin resolved upon its discontinuation. No patients developed renal or hepatic impairment as a result of taking gabapentin.

The average OMWQ-HN mouth and throat soreness ratings (scored out of 10) during the last week of RT for the 3600 mg gabapentin group, the 300–3200 mg group, and the 0 mg group were 5.38 (95% CI: 5.01–5.75), 5.31 (95% CI: 4.88–5.74), and 5.78 (95% CI: 5.27–6.29), respectively. According to Fisher’s exact test there was no difference among the groups regarding the development of severe OM (*p* = 0.61).

**Figure 1:**
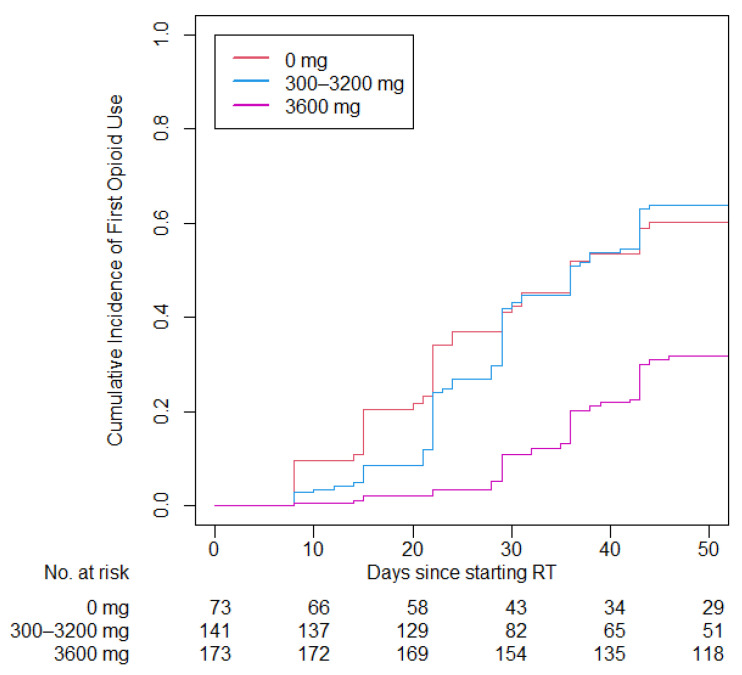
Cumulative incidence of first opioid use during RT over time.

Patients on opioids prior to the start of RT (93 patients, 19.4%) were excluded from the analysis of opioid use during RT and time to first opioid use. The multivariate competing risk model was adjusted for sex, race, age, performance status, smoking status, primary disease site, cancer stage, HPV status, treatment type, and chemotherapy type. The time to first opioid use was greater in the 3600 mg gabapentin group compared with the no gabapentin group (34.3 vs. 23.9 days; *p* < 0.001) and compared with the 300–3200 mg group (34.3 vs. 28.0 days; *p* < 0.001; Figure 1). The adjusted hazard ratio was 3.17 (95% CI: 2.08–4.83; *p* < 0.001) for the 0 mg cohort compared with the 3600 mg cohort and 2.65 (95% CI: 1.89–3.72; *p* < 0.001) for the 300 to 3200 mg cohort compared with the 3600 mg cohort. The proportion of patients requiring opioids at any point during RT was lower in the 3600 mg gabapentin group compared with the no gabapentin group (31.8% vs. 60.1%; *p* < 0.001) and compared with the 300–3200 mg group (31.8% vs. 63.8%; *p* < 0.001).

An analysis of the FT insertion rate was performed on all definitive RT patients who received RT from 2017 to 2022 (*n* = 263). Multivariate logistic regression analysis demonstrated the 3600 mg gabapentin group did not have significantly reduced odds of FT placement compared with the no gabapentin group (14.3% vs. 25.0%, adjusted OR: 1.38; 95% CI: 0.39–4.45; *p* = 0.60), nor was it significant for the 300 to 3200 mg group compared with the no gabapentin group (23.5% vs. 25.0%, adjusted OR: 1.42; 95% CI: 0.63–3.22; *p* = 0.39).

## 4. Discussion

Prophylactic use of 3600 mg gabapentin was well tolerated, halved the overall opioid use, and significantly delayed the time to first opioid use for OM pain management during RT while maintaining similar patient-reported OM soreness scores. Consistent with our study, Smith et al. found that gabapentin decreased pain during RT; however, they were unable to escalate most patients beyond 900 mg, while 86% of patients without contraindications for gabapentin tolerated escalation to 3600 mg in our study [5]. A prospective randomized trial examining the addition of venlafaxine to 3600 mg gabapentin found no improvement in pain control or quality of life but similarly demonstrated that more than 90% of patients in the trial tolerated high-dose gabapentin through to the conclusion of treatment [13]. Our tolerability results are consistent with a large multicenter study of 2216 patients that found that only 10.6% of patients prematurely discontinued gabapentin due to adverse events [14].

Similar to our results, a previous retrospective study found that only 35% of HNSCC patients required opioids for pain control during the last weeks of RT when a median dose of 2700 mg gabapentin daily was given [15]. In a placebo-controlled randomized trial, Cook et al. found no benefit to 1800 mg of gabapentin daily, and, surprisingly, patients on gabapentin had a higher rate of FT placement (62.1% vs. 20.7%, *p* = 0.01) [6]. In our study, 3600 mg gabapentin nominally reduced the FT rate compared with those not taking gabapentin, but this was not statistically significant. Several other studies have found gabapentin use to be associated with lower FT rates [16,17,18]. The cumulative incidence of opioid use over time did not vary in the no gabapentin group versus the 300–3200 mg gabapentin group, for which the median dose was 1800 mg, suggesting that 1800 mg of gabapentin may not be a high enough dose to be efficacious.

The major limitations of our study include its retrospective nature, particularly the fact that most 3600 mg gabapentin patients were treated after July 2018. Patients in the cohort taking 3600 mg of gabapentin were less likely to be current smokers and to have an oral cavity primary, both of which are associated with the development of oral mucositis [19,20,21]. Multivariate analyses were performed to adjust for such potential confounders. Additionally, it is important to acknowledge that gabapentin can cause adverse side effects such as somnolence (15.2%), dizziness (10.9%), and asthenia (6.0%) [14]. Nevertheless, prophylactic 3600 mg gabapentin was well tolerated and halved the proportion of patients requiring opioid use at any time during RT while maintaining similar OM soreness scores.

## 5. Conclusions

We report that in a large observational cohort, prophylactic use of 3600 mg gabapentin was well tolerated, halved overall opioid use, and delayed the time to first opioid use during radiation therapy. Multi-institutional prospective studies are warranted to investigate the efficacy and tolerability of high-dose gabapentin in OM pain control.

## Figures and Tables

**Table 1:** Clinical characteristics of the overall cohort.

	No. (%)				
Characteristic	Total Cohort	Gabapentin, 0 mg	Gabapentin, 300 to 3200 mg	Gabapentin, 3600 mg	*p*-Value
Total patients	480	112	182	186	
Age, mean (SD), year	62.5 (9.6)	62.0 (8.6)	62.0 (9.3)	61.9 (8.6)	0.66
Sex					
Male	370 (77.1)	82 (73.2)	145 (79.7)	143 (76.9)	1.00
Female	110 (22.9)	30 (26.8)	37 (20.3)	43 (23.1)	
Race					
White	435 (90.6)	97 (86.6)	170 (93.4)	168 (90.3)	0.63
Other ^a^	45 (9.4)	15 (13.4)	12 (6.6)	18 (9.7)	
Karnofsky performance status					
≥80	415 (86.5)	91 (81.3)	162 (89.0)	155 (83.3)	0.01
<80	65 (13.5)	21 (18.8)	20 (11.0)	24 (12.9)	
Tobacco status					
Never	133 (28.0)	22 (19.6)	49 (26.9)	62 (33.3)	0.04
Former	279 (58.1)	71 (63.4)	102 (56.0)	106 (57.0)	
Current	68 (14.0)	19 (17.0)	31 (17.0)	18 (9.7)	
T-stage					
T0	21 (4.4)	4 (3.6)	12 (6.6)	5 (2.7)	0.64
T1	69 (14.4)	14 (21.5)	25 (13.7)	30 (16.1)	
T2	144 (30.0)	36 (32.1)	55 (30.2)	53 (28.5)	
T3	155 (32.3)	32 (28.6)	64 (35.2)	59 (31.7)	
T4	91 (19.0)	26 (23.2)	26 (14.3)	39 (21.0)	
N-stage					
N0	101 (21.0)	27 (24.1)	37 (20.3)	37 (19.9)	0.19
N1	134 (27.9)	26 (23.2)	38 (20.9)	70 (37.6)	
N2	198 (41.3)	50 (44.6)	92 (50.5)	56 (30.1)	
N3	47 (9.8)	9 (8.0)	15 (8.2)	23 (12.3)	
HPV status					
Negative	82 (17.1)	26 (23.2)	34 (18.7)	22 (11.8)	0.06
Positive	232 (48.3)	50 (40.6)	88 (48.4)	94 (50.5)	
Unknown	166 (34.6)	36 (32.1)	60 (33.0)	70 (36.1)	
Disease site					
Larynx	88 (18.3)	23 (20.5)	28 (15.4)	37 (19.9)	0.01
Oropharynx	245 (51.0)	53 (47.3)	98 (53.8)	94 (50.5)	
Nasopharynx	16 (3.3)	5 (4.5)	4 (2.2)	7 (3.8)	
Hypopharynx	26 (5.4)	6 (5.4)	9 (4.9)	11 (5.9)	
Lateral neck	22 (4.6)	5 (4.5)	4 (2.2)	13 (7.0)	
Oral cavity	83 (17.3)	20 (17.9)	43 (23.6)	20 (10.8)	
Treatment type					
CCRT	292 (60.8)	56 (50.0)	128 (70.3)	108 (58.1)	0.50
Surgery + RT	49 (10.2)	16 (14.3)	16 (8.8)	17 (9.1)	
ICT + CCRT	32 (6.7)	7 (6.3)	14 (7.7)	11 (5.9)	
RT only	29 (6.0)	11 (9.8)	7 (3.8)	11 (5.9)	
Surgery + CCRT	78 (16.3)	22 (19.6)	17 (9.3)	39 (20.1)	
Type of chemotherapy					
Cisplatin	325 (67.7)	70 (62.5)	124 (68.1)	131 (70.4)	0.31
Other	155 (32.3)	42 (37.5)	58 (31.9)	55 (29.6)	
Type of RT					
Definitive	358 (74.6)	76 (67.9)	148 (81.3)	134 (72.0)	0.28
Post-operative	122 (25.4)	36 (32.1)	34 (18.7)	52 (28.0)	
Feeding tube used					
Yes	165 (34.4)	52 (46.4)	63 (34.6)	50 (26.9)	<0.01
No	315 (65.6)	60 (53.6)	119 (65.4)	136 (73.1)	
Hospitalized during RT					
Yes	108 (22.5)	21 (18.8)	45 (24.7)	42 (22.6)	0.43
No	372 (77.5)	91 (81.3)	137 (75.3)	144 (77.4)	

Abbreviations: No.: number; SD: standard deviation; KPS: Karnofsky performance status; CCRT: concurrent chemoradiation; RT: radiation therapy; ICT: induction chemotherapy. ^a^ Other in the Race category included Asian or Pacific Islander, Black or African-American, and unreported/unknown (patients declined to report their race).

## Data Availability

Research data are stored in an institutional repository and will be shared upon request to the corresponding author.
